# EFL/ESL Students' Perceptions of Distributive, Procedural, and Interactional Justice: The Impact of Positive Teacher-Student Relation

**DOI:** 10.3389/fpsyg.2021.755234

**Published:** 2021-09-28

**Authors:** Dan Yang

**Affiliations:** Foreign Language School, Yancheng Teachers University, Yancheng, China

**Keywords:** teacher-student interpersonal communication, distributive justice, procedural justice, interactional justice, teacher-student relation

## Abstract

The correlation between teacher-student interpersonal relationships and students' perception of different dimensions of justice using in the learning context has been found absolutely important since it can provide a nice learning environment for students in which they can comfortably learn a new language. Even though several studies have been carried out regarding the above-mentioned points, a review paper that focuses on the importance between these two variables by which students' learning is influenced seems of great interest. In this study, the author has strived hard to highlight the interplay between the aforementioned variables. First of all, Justice and its dimensions including distributive, procedural, and interactional justice are described in the learning context. Then the effect of the positive relationship between teachers and students is accentuated. Following it, different types of characteristics that are crucially noticeable considering teacher-student interpersonal relationship including “teachers care,” “teacher clarity,” “teacher confirmation,” “teacher credibility,” “teacher immediacy,” “teacher stroke,” “teacher-student rapport” are discussed. The term “positive psychology” accompanied by its factors is defined then. What is discussed then is classroom justice as a teacher-student interpersonal factor. Finally, it is concluded with implications and suggestions for future studies.

## Introduction

The interpersonal relationship between teachers and students has always been highlighted in different studies in that it is a mutual relationship, and it has been proposed that care should be given to enhance such a relationship between teachers and students; as a consequence, with regard to the positive psychology, diverse types of characteristics in teachers should be stressed and enhanced if needed to take advantage of the learning context (Xie and Derakhshan, 2021). Moreover, varied dimensions of justice have been highlighted in some studies since for a just educational context, it seems worthwhile (Estaji and Zhaleh, 2021). The factors of positive psychology such as well-being and emotion regulation have been mentioned in some studies to help teachers enhance their mood and have a better understanding of how they should act during the class which enables them to be better versions of themselves (Wang et al., 2021). In this study review, information was collected to emphasize the impact of students' perception of various dimensions of justice including distributive, procedural, and interactional justice, in association with the teacher-student interpersonal relationship. Firstly, justice and its dimensions including distributive, procedural, and interactional justice have been defined in the learning context. Then, the impact of the positive relationship between teachers and students has been highlighted. Following it, different types of characteristics that are crucially important regarding teacher-student interpersonal relationships have been discussed. The term “positive psychology” accompanied by its factors has been conceptualized then. Classroom justice as a teacher-student interpersonal factor has been taken into consideration. Finally, it has been concluded with implications and suggestions for future studies.

## Background

### EFL/ESL Students' Perceptions of Different Types of Justice

Justice is essentially important in everyone's life; it is applied in society both politically and socially and it can also be seen in organizational behavior. It is viewed as a value since many other factors depend on it in society (Cropanzano and Greenberg, 1997). Social psychology theory of justice has been established in the learning context by some key figures. It has been found that many student variables are influenced by students' understanding of classroom justice such as the student' being motivated, achieving academic goals, engagement, the relationship between teachers, and students, reactions which are behavioral or emotional, showing interest in the subjects that are shared through the class, the amount of willingness to talk, feeling about the teacher, and learning cognitively (Estaji and Zhaleh, 2021).

#### Distributive Justice

Organizational justice is defined as the evaluation of one's fairness in the organizational processes, consequences, and communications (Kazemi et al., 2015). This type of justice can be classified into three categories: distributive, procedural, and interactional justice. Distributive justice is conceptualized as an understanding of fairness considering distributing outcomes. Three following principles can clarify this type of justice more: need (how the outcome is distributed when one expects or needs something), equity (how the outcome is distributed according to one's perseverance, contribution, and performance), and equality (how equally outcome among people are distributed) (Deutsch, 1975).

#### Procedural Justice

Procedural justice refers to the understanding of fairness in terms of utilized measures and policies so as to make allocation decisions. This justice is viewed to be kept when the measures are judged to be fair such as “bias dominance principle,” recognized on satisfactory and accurate information such as “accuracy principle,” engaged regularly across time and individuals such as “consistency principle,” adaptable such as “correctability principle,” considering all individuals' concerns who are engaged such as “voice principle,” resting on the predominant ethical and moral values such as “ethicality principle,” enacted clearly and with clarity such as “transparency principle,” and are rational “reasonableness principle” (Leventhal, 1980; Kazemi and Törnblom, 2008; Rasooli et al., 2019).

#### Interactional Justice

Interactional justice, the third classification of organizational justice, relates to the understanding of fairness in conveying information and interpersonal relationships when individuals perceive to be in a friendly atmosphere such as “caring principle,” behaved reverentially such as respect principle, and with dignity such as “propriety principle,” and when information is transferred to them in an appropriate manner such as “timeliness principle,” sincerely such as “truthfulness principle,” and according to sufficient and rational clarifications such as “adequacy/justification principle,” (Greenberg, 1993; Colquitt, 2001; Rasooli et al., 2019). Out of the three dimensions, distributive justice is the most crucially important from teacher's point of view which can be seen in different forms, from grading, giving feedback to praising, and providing students with opportunities (Ciuladien and Račelyte, 2016). The distributive justice has been said to be the most important since in the next two classifications of justice, the focus is shifted to students themselves: procedural justice including how students' homework is evaluated, what methods are used by the teacher to manipulate the class, and the strategies applied in the class to control students' behavior, and instructional justice that is concerned with how much students are treated reverently and politely by the teachers, and if the information can be clearly conveyed to the students by the teacher (Ciuladien and Račelyte, 2016). Distributive justice concentrates on the grades which students receive and on students who stand out enough to be noticed by the teacher and attract teachers' attention (Chory-Assad and Paulsel, 2004).

### The Importance of Positive Teacher-Student Relation

Before, the relationship between some negative factors such as anxiety and the teaching-learning context has been discussed (Seifalian and Derakhshan, 2018). Much attention has been focused on positive psychology in the pedagogical contexts for the last two decades. Positive behaviors between EFL teachers and students are perceived to have a paramount impact on students' achievements. Such behaviors can be considered either negative or positive. According to Xie and Derakhshan (2021) “teacher care, clarity, credibility, rapport with students, stroke, immediacy, and confirmation” expound how teachers positively anticipate the academic outcomes such as “motivation, learning, engagement, involvement, class attendance, willingness to communicate, performance, and success, p. 1” in students. Therefore, the above-mentioned factors in teachers can be viewed as positive which causes students to feel fulfilled and allow their wants and needs to be met (Frymier, 2016; Goldman et al., 2017). As the name suggests, positive psychology made a shift from a regular approach to life to a more open-minded outlook on life; as a result, positive emotions have been fostered in students if there is a positive, productive relationship between the teachers and students (Wang et al., 2021). It has been corroborated that students are more likely to think of their teachers as just if they have been provided with supportive interactions in the classroom in which the students' needs can be met (Gasser et al., 2018). Teacher care was first introduced by Noddings (1984) which refers to the amount of empathy shared and the openness in face of other people's needs. Likewise, the same goes for the educational contexts (Gasser et al., 2018). It is claimed that when students are conscious of teachers' care toward themselves during the class, they are more likely to feel secure (Noddings, 2006). Students' well-being, feeling revered, the amount of being engaged during the class, the level of self-esteem, and their performance are stimulated when teachers show care (Derakhshan et al., 2019).

When people try to convey their meaning and information more understandable, it is where we consider clarity and transparency (Myers et al., 2014). From an institutional point of view, it refers to teachers when they try to clarify something by giving more examples, using visual aids, utilizing verbal language, and repeating something (Violanti et al., 2018). Teacher clarity leads to students processing and retrieving what they have been taught in a better way since when they are taught, the students make an endeavor to transfer the information from the working memory to the long-term memory (Bolkan, 2017). Confirming communications make students feel heard, recognized, and valued (Ellis, 2000; Burns et al., 2018). When students are provided with feedback, it increases their enthusiasm about learning and it is what makes the learning contexts much more enjoyable and causes an excellent rapport to be shaped between teachers and students (Ellis, 2000; Goldman et al., 2014). It has been told that students feel more satisfied and their level of willingness to talk will raise if they are given confirming answers or feedback by their teachers (Sidelinger and Booth-Butterfield, 2010). In the education domain, teacher credibility refers to the extent to which a teacher is credible or trustworthy (Teven and McCroskey, 1997). It has been unraveled that students' willingness to attend the class increases when the students trust in their teachers (Pishghadam et al., 2019). Within the instructional context, teacher immediacy is concerned with verbal and non-verbal prompts diminishing the psychological or physical distance between teachers and students (Estepp and Roberts, 2015). Verbal immediacy can be exemplified in this way: when students are asked about their ideas, they are asked to be involved in a friendly conversation, and teachers use a great sense of humor. Non-verbal immediacy, on the other hand, refers to teachers smiling, making eye contact, and using relaxing postures (Wendt and Courduff, 2018).

Strokes can be classified into six groups: positive such as “what nice clothes,” negative such as “I loathe you,” verbal such as “how are you?,” non-verbal such as smiling, conditional such as “you are a nice person,” or unconditional such as “I love you.” It has been indicated that there is a positive association between teacher stroke and teacher factors such as teacher success, credibility (Pishghadam et al., 2021), care, and conceptions of intelligence (Derakhshan et al., 2019). Rapport is the relationship between teachers and students which is characterized by reverence, enjoyment, and shared trust (Frisby and Martin, 2010; Frisby and Housley Gaffney, 2015). Rapport can be seen in the following forms throughout the classroom: when humor is used by teachers, when appropriate feedback is provided, when students' attitudes are revered, and when enthusiasm is expressed in students' learning (Weimer, 2010).

### Positive Psychology Factors

Positive psychology has increasingly been growing in L2 education and in 2016, two books were written by MacIntyre et al. (2016) and Gabry's-Barker and Gałajda (2016) which put more emphasis on this great line of research. MacIntyre and Gregersen (2012) conceptualized foreign language enjoyment that is defined as positive emotion arising after a goal has been achieved. It leads to students broadening their horizons, being more engaged in the learning process, and achieving better academic results (Pekrun, 2006). It is regarded as a feeling of pleasure when one completes a task successfully and appreciates the content of learning. Not only does it have a good impact on the students but it also affects teachers in a way that it causes them to overcome anxiety (Dewaele and Dewaele, 2020). Research shows that factors related to teachers play a more crucial role than factors which are related to learners. Hence, variables such as being supported by the teachers, using a great sense of humor, being friendly with the students, respecting students, using a nice tone, and sharing positive moods are amongst the positive variables relevant to teachers that can increase enjoyment.

Another factor relevant to positive psychology is well-being (Oxford, 2016). Well-being is described as how satisfied a person is with his mental health, life, and work (Garg et al., 2014). Ryff (1989) mentioned that well-being is associated with accepting oneself, growing personally, having aims in life, being autonomous, and having environmental mastery.

Resilience is another intrapersonal factor that should be kept in mind in that it enhances one's productivity when confronting with difficult circumstances on a daily basis. It is defined as the capacity to adapt to unpleasant situations with which they are faced (Bobek, 2002). Resilience is linked to various L2 educational consequences such as motivation for learning, teaching satisfaction, and well-being.

The fourth factor in the field of positive psychology is emotion regulation that is described as extrinsic and intrinsic processes that one faces to assess, adjust, or control his emotions to achieve particular goals in life (Proietti Ergün and Dewaele, 2021). When teachers or students know how to regulate their emotions either positive or negative skillfully in the classroom, it highly affects the relationship between teachers and students, what students gain through the learning process, and teachers' success (Ghanizadeh and Moafian, 2010; Teng and Zhang, 2016).

The last factor in the realm of positive psychology is academic engagement that is conceptualized as cognitive, emotional, and behavioral dimensions (Reschly and Christenson, 2012). Behavioral engagement pertains to learners' authentic tendency to partake in the activities or tasks (Mercer, 2019). Cognitive engagement occurs when a person is sufficiently and mentally challenged and immersed in one's work. Emotional engagement is perceived as students' feelings of dedication and attachment to a task (Reschly and Christenson, 2012). According to the studies conducted so far, students' L2 engagement was discovered to be positively associated with teacher care, rapport, non-verbal immediacy, and credibility behaviors (Derakhshan, 2021), emotions, grit (Khajavy, 2021), enjoyment (Dewaele and Dewaele, 2020), willingness to communicate and bilingual learning (Mystkowska-Wiertelak, 2021).

Grit as another factor that is concerned with perseverance, desire, and energy for long-term goals (Duckworth et al., 2007). One of the attributes of grit is malleability, meaning that it can be boosted through involvement and education in the instructional context (Clark and Malecki, 2019). In the classroom, using malleability of grit, learners can be prepared for the possible difficulties and challenges when learning a new language. It has been said that there is a positive association between academic results, willingness to communicate, and enjoyment (Akos and Kretchmar, 2017).

In terms of educational context, love is defined as a learning experience that is meaningful and positive. Learners' emotional and social development is greatly affected by love (Loreman, 2011). It has been asserted that successful learning occurs when there is a loving, friendly context (Yin et al., 2019). Love has also been emphasized in Maslow's (1954) hierarchy of needs. Self-actualization, which puts on top of the pyramid, is achieved when the need for love is fulfilled. So in the realm of learning, only when students' needs are met, does success happen. To this end, students should be respected, supported, and understood by their teachers. A loving teacher is capable to boost students' functioning. It should be taken into consideration that efficacious coping mechanisms can be created and students can feel motivated when love exists (Pavelescu and Petri'c, 2018).

### Classroom Justice as a Teacher-Student Interpersonal Factor

In terms of instructional context, classroom justice is defined as how students evaluate the fairness of outcomes and processes (Chory-Assad and Paulsel, 2004). When it comes to distributive justice, “outcome” pertains to the grades which are received by the students. Students' perception of interactional justice is highly correlated with the respect they receive from their teachers and the amount of openness toward their opinions which is interpreted as teacher care or teacher stroke contributing to better well-being in both students and teachers (Chory, 2007). Students consider those teachers who do not admit their ideas and hurt their feelings by giving negative feedback about their performance as unfair. It was claimed that the teacher-student rapport and teacher confirmation have played a crucial role in students' perception of justice so far. Ashworth et al. (1997) on the qualitative research methods course unit discovered that students' understanding is affected by the way they are graded by their professors for example for group work. According to a study conducted by Chory et al. (2014) teachers are highly likely to be viewed as unfair by the students when the students are treated badly about their concerning grades. The better the students are treated by the teachers and the more productive the feedback is, the more the teachers can be viewed as trustworthy that has been mentioned earlier, teacher credibility. It was said that more than half of the unjust behaviors reported by the students were relevant to grades. Additionally, opposition to authority, which was followed by verbal hostility and disengagement was also of paramount importance. It was additionally predicted the relationship between students' understanding of teachers' unfairness and their behavioral responses is mediated by students' emotional responses to unfairness. The way injustice is perceived by the students can cause an increase in caustic behavior, fight, dishonesty, anger, and conflict in the classroom which blemishes the relationship between teachers and students and also gives rise to a deep hatred toward teachers (Ciuladien and Račelyte, 2016).

A study carried out by Estaji and Zhaleh (2021) indicated that in terms of distributive justice, students believe that students' affective and cognitive and learning are directly impacted by justice. Additionally, teachers play a tremendous role in students' learning. Thus, had teachers been more careful about justice, the students would not have felt upset, demotivated, and discouraged leading to students distrusting their teachers. Students also claimed that students should be treated equally and the learning opportunities should equally be provided by fair teachers. To put it simply, it makes a bond of trust and understanding between teachers and students. Based on what students put forward, another factor which makes a teacher fair was that they make eye contact from time to time with students that can be described as teacher immediacy and teacher stroke. On the exam day, what attracts attention is that fair teachers should give help to all the students equally and the same amount of feedback should be given to students. Individual differences are what should be taken into consideration and as a result, the way the subject-matter is taught by the teachers should vary from learner to learner due to the fact that they have various learning styles. In terms of homework assignments, students should be provided with different types of homework based on their abilities. Depending on students' character, being extroverted or introverted, they are expected to behave in a different way and as result, the students are treated differently by the teachers that can be perceived as positive or negative and it may boost or spoils the intrapersonal relationship between students and teachers. It was also claimed by the students that some students are well-behaved and grateful that causes a good rapport to be built up with the teachers, whereas other students do not revere the rules of the class and its value. Accordingly, should teachers behave the students in this way, it may reinforce negative behaviors. In terms of procedural justice, a fair teacher is not impartial when a student is absent and is not strict toward another one. There should be no differentiation among students when a rule is broken. As it has been mentioned above, teacher confirmation either negative or positive highly affect students' learning. Another feature for fair teachers is that students' ideas are of great importance when a date should be fixed for taking a test and a topic should be determined for discussion, for instance. Standards and rubrics are planned by fair teachers so that students perceive what they are expected to do throughout the class, leading to students having a better image of their teachers. In terms of Interactional justice, students are treated in a friendly way by just teachers that can be shown in term “teacher stroke or teacher immediacy.” A positive, safe, friendly atmosphere is created by just teachers in the classroom. Similarly, students' poor performance is not belittled and humiliated by just teachers that completely ruins the relationship between the students and teachers. Consequently, the students are addressed respectfully by just teachers.

## Implications and Further Suggestions

Studies that have been carried out considering students' perception of justice and positive interactional teacher-learner relationships are countless. It is of great importance to analyze how students feel about the realm of learning education and how the class and every single detail of it can be interpreted in their minds since learners are claimed to play a paramount role in the learning context. The more the students are satisfied with the learning context, the more valued teachers feel and as a result, a loving, friendly atmosphere can be created so that students can achieve their goals. Regarding the above-mentioned points, the focus of this study was the students' perception of different types of justice within the interpersonal teacher-learner relationship. Despite the fact that positive psychology has attracted much attention to itself over the last two decades, some topics such as the one studied in this research needs to be given more attention since students' interpretation of the classroom is of crucial significance. Further studies that can be done by avid researchers are classified into five categories: first and for most, one of the components of positive psychology is loving pedagogy; therefore, studies should be done so as to find the correlation between students' perception of various types of justice and a loving learning context to see how they affect each other. Secondly, even though students' perception is found to be the most important of all, teachers' perception is also extremely crucial since they are supposed to manage the class and take control of their feelings in order to allow students to enjoy a friendly atmosphere of the class and subject-matter. Thirdly, a longitudinal study should be conducted in which different types of justice can be explicated for the students and their effect on the learning process can be studied since the only way through which the infrastructures for academic standards can be strengthened is trying to build a learning atmosphere in which students are treated in just manner. Amongst the three dimensions of justice, the most significant one is distributive justice in the teachers' perceptions. Because it is a necessity for teachers to be cognizant of grading the students, rewarding the students, giving feedback to all students, praising the students, giving equal opportunities to students fairly. However, the least accentuated justice was interactional justice out of the three dimensions of justice. It can clarify it for teachers that being fair in interacting and conveying the meaning to students is not as significant as being just in interactions and being fair when outcomes are distributed and applying class procedures. Fourthly, variables such as age can play a paramount role in the way teachers perceive different conceptions like justice; consequently, this variable can be controlled in further studies to see the difference in how justice is conceived by teachers from different age groups. finally, in the field of positive psychology, a point which is regarded as encouraging is that applying such studies in real learning contexts can help the educational domain no end in that students' engagement and performance can be enhanced and high educational standards can be exploited that help both teachers and learners to enjoy the teaching and learning contexts respectively.

It should be concluded that different types of people can take advantage of the current review study and needless to say, the authorities, teachers, and students are not exceptions. One of the characteristics of quality teachers is putting justice into practice in their classes and it is said that it would be considered their responsibility to apply justice practices in their classes. Consequently, teachers can be enticed by policymakers to ameliorate the way they use justice in their teaching methods and their behaviors. Another point that is of pivotal importance for policymakers from an institutional point of view is that teachers' characteristics are changeable regarding a better educational system, so in an effort to modify their attributes, teachers should be trained how to develop a sense of responsibility and confidence in themselves to heighten their performance in the classroom.

## Author Contributions

The author confirms being the sole contributor of this work and has approved it for publication.

## Conflict of Interest

The author declares that the research was conducted in the absence of any commercial or financial relationships that could be construed as a potential conflict of interest.

## Publisher's Note

All claims expressed in this article are solely those of the authors and do not necessarily represent those of their affiliated organizations, or those of the publisher, the editors and the reviewers. Any product that may be evaluated in this article, or claim that may be made by its manufacturer, is not guaranteed or endorsed by the publisher.
